# Discordance of Apolipoprotein B, Non-HDL-Cholesterol, and LDL-Cholesterol Predicts Risk of Increased Arterial Stiffness and Elevated Carotid Intima-Media Thickness in Middle-Aged and Elderly Chinese Adults

**DOI:** 10.3389/fcvm.2022.906396

**Published:** 2022-05-18

**Authors:** Xiaojing Jia, Yan Qi, Ruizhi Zheng, Lin Lin, Chunyan Hu, Yuanyue Zhu, Qiuyu Cao, Xueyan Wu, Hongyan Qi, Ran Wei, Yi Zhang, Min Xu, Yu Xu, Tiange Wang, Zhiyun Zhao, Yuhong Chen, Mian Li, Weiqing Wang, Yufang Bi, Jieli Lu

**Affiliations:** ^1^Department of Endocrine and Metabolic Diseases, Shanghai Institute of Endocrine and Metabolic Diseases, Ruijin Hospital, Shanghai Jiao Tong University School of Medicine, Shanghai, China; ^2^Shanghai National Clinical Research Center for Metabolic Diseases, Key Laboratory for Endocrine and Metabolic Diseases of the National Health Commission of the PR China, Shanghai Key Laboratory for Endocrine Tumor-State Key Laboratory of Medical Genomics, Ruijin Hospital, Shanghai Jiao Tong University School of Medicine, Shanghai, China

**Keywords:** discordance, apolipoprotein B, non-high-density lipoprotein cholesterol, low-density lipoprotein cholesterol, arterial stiffness, carotid intima-media thickness

## Abstract

**Background:**

Apolipoprotein B (apoB) and non-high-density lipoprotein cholesterol (non-HDL-C) have been shown to predict cardiovascular disease (CVD) even in the case of low levels of low-density lipoprotein cholesterol (LDL-C). We aimed to investigate whether the discordance between LDL-C and apoB or non-HDL-C was associated with arterial stiffness and elevated carotid intima-media thickness (CIMT) in middle-aged and elderly adults.

**Methods:**

A total of 5,279 Chinese adults free of CVD at baseline were included and followed with a mean follow-up of 4.3 years. Arterial stiffness was measured by brachial-ankle pulse wave velocity (baPWV) and pulse pressure (PP). The associations of apoB, non-HDL-C, and LDL-C with arterial stiffness or elevated CIMT were examined with logistic regression models using either continuous scales by restricted cubic splines or categories of concordant and discordant values defined by medians.

**Results:**

High apoB but not LDL-C was associated with elevated baPWV or PP. High apoB, non-HDL-C, and LDL-C were all associated with elevated CIMT (*p* < 0.05). Individuals with low levels of LDL-C and discordantly high apoB or non-HDL-C compared to those with concordantly low apoB or non-HDL-C demonstrated higher risks of elevated baPWV [ORs (95% CI) of 1.40 (1.03–1.91) and 1.56 (1.12–2.18), respectively] and elevated PP [ORs (95% CI) of 1.61 (1.19–2.18) and 1.55 (1.12–2.15), respectively]. While, discordant high LDL-C with low apoB was associated with an increased risk of elevated CIMT (OR, 1.74; 95% CI, 1.13–2.69).

**Conclusion:**

Discordance analysis revealed that elevated apoB or non-HDL-C was a better predictor of risk of arterial stiffness, whereas LDL-C for elevated CIMT.

## Introduction

Cardiovascular diseases (CVDs) remain the leading cause of health loss throughout the world (1). Multiple risk factors have been identified for CVDs, among which abnormal lipids account for a considerable proportion (2). Low-density lipoprotein cholesterol (LDL-C), an extensively studied lipid trait, has been recognized not only as a risk predictor but a causal factor for CVD (3). Definite reductions in the incidence of major vascular events could be obtained by lowering cholesterol, especially relying on the reduced number of LDL particles (4). However, a prospective meta-analysis shows that one in seven patients with cholesterol-lowering treatment had CVD events over 5 years (5). The existed “residual risk” among individuals with low LDL-C is partly attributed to an undeniable role of other lipid particles.

Samples from either the general population or large clinical studies show that a certain percentage of individuals have non-high-density lipoprotein cholesterol (non-HDL-C) or apolipoprotein B (apoB) above the recommended targets in those with low LDL-C (6). Non-HDL-C encompasses the cholesterol information of atherogenic lipid particles [LDL, very low-density lipoprotein (VLDL), intermediate-density lipoprotein (IDL), and lipoprotein (a)]. There is exactly one molecule apoB carried on the surface of each atherogenic lipid particle (7). ApoB provides a direct way to estimate the total number of these lipoproteins. Both apoB and non-HDL-C are proven to have a relation to CVD (8, 9). Meanwhile, Johannesen et al. have demonstrated that populations with LDL-C below the median but apoB or non-HDL-C above present increased risks of myocardial infarction [ORs (95% CI) of 1.49 (1.15–1.92) and 1.78 (1.35–2.34), respectively] and all-cause mortality [ORs (95% CI) of 1.21 (1.07–1.36) and 1.18 (1.02–1.36), respectively] compared with concordant low values (10), suggesting that such two lipid traits may be a better driver of cardiovascular risk rather than LDL-C alone.

Previous studies revealed that elevated carotid intima-media thickness (CIMT) and arterial stiffness measured by elevated brachial-ankle pulse wave velocity (baPWV) or elevated pulse pressure (PP) were all accompanied by a higher risk of CVD (11–13). These measurements are all reckoned as a prelude and a great threat to CVD health. Early detection and preventive intervention on subclinical cardiovascular events are crucial to reducing the CVD risk. However, few prospective studies evaluate the predictive values of discordant lipids in association with arterial stiffness and elevated CIMT among middle-aged and elderly adults.

To fulfill this knowledge gap, we conducted a discordance analysis on the associations of apoB, non-HDL-C, and LDL-C with the risk of elevated baPWV, elevated PP, or elevated CIMT in a community-based cohort study.

## Materials and Methods

### Study Population

**FIGURE 1:**
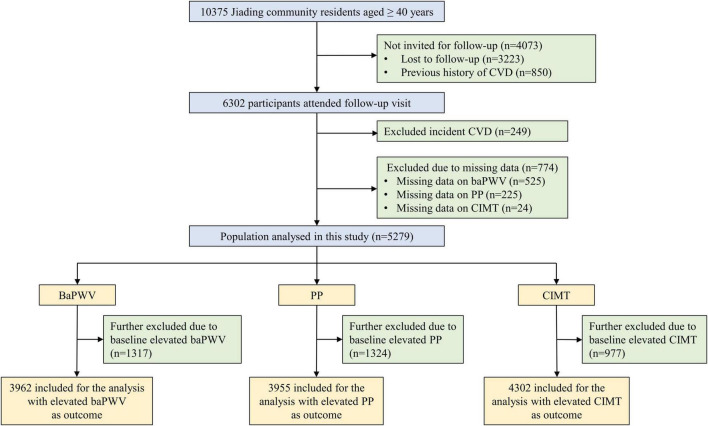
Flowchart of study procedure. CVD, cardiovascular disease; BaPWV, brachial-ankle pulse wave velocity; PP, pulse pressure; CIMT, carotid intima-media thickness.

The middle-aged and elderly study participants were from a prospective population-based cohort in Jiading District, Shanghai, China. The design and eligibility criteria of the overall study had been previously described in detail elsewhere (14). Briefly, the study was launched between March and August 2010 among 10,375 registered permanent residents aged 40 years or older, who all underwent a comprehensive survey comprising of a standard questionnaire and relevant biochemical measurements. For the current study, we excluded individuals who had a previous history of CVD (*n* = 850), defined as a composite endpoint of fatal or non-fatal myocardial infarction, stroke, hospitalization, or treatment for heart failure. From August 2014 to July 2015, all eligible participants were invited to attend a follow-up visit and 6,302 individuals complied. Given the investigated outcomes was an early stage of CVD, we further excluded 249 participants who developed CVD during the 5.4 years of follow-up (mean 4.3 years) to avoid influencing or even overestimating the target risk. Participants without baseline or follow-up information on baPWV, PP, or CIMT were not included, leaving 5,279 for this analysis. In addition, we excluded participants with baseline elevated baPWV (*n* = 1,317), elevated PP (*n* = 1,324), and elevated CIMT (*n* = 977) from analysis for each outcome. A detailed flowchart of the study is presented in Figure 1.

The study protocol conformed to the Declaration of Helsinki and had been approved by the Institutional Review Board of the Ruijin Hospital, Shanghai Jiao Tong University School of Medicine. All participants provided written informed consent.

### Data Collection and Clinical Evaluation

A standardized, interviewer-administered questionnaire was used to collect information on demographic characteristics, current use of medications and lifestyle factors, etc. Current drinkers or smokers were defined as those who consumed alcohol once per week or smoked cigarettes once per day regularly during the past 6 months, respectively. We defined ideal physical activity as moderate-intensity ≥ 150 min/week or vigorous-intensity ≥ 75 min/week or moderate and vigorous-intensity ≥ 150 min/week using the International Physical Activity Questionnaire (15).

Each study participant was measured for body weight, height, and systolic and diastolic blood pressure (BP). Body mass index (BMI) was calculated as body weight (kg) divided by the square of body height (m). BP was measured three times using a calibrated automated electronic device (OMRON Model HEM-725 FUZZY, Omron Company, Dalian, China) on the non-dominant arm of seated participants who had rested after 5 min. The mean value of three BP readings was calculated for analysis. Pulse pressure (PP) was equal to systolic BP (SBP) minus diastolic BP (DBP).

BaPWV was determined at both baseline and follow-up by trained technicians using Colin VP-1000 [Model BP203RPE II, form PWV/ABI (ankle-brachial index); OMRON Colin Medical Instruments, Tokyo, Japan]. Pulse waves from both sides of the upper arms and ankles were obtained simultaneously through cuffs. The right and left baPWV were calculated by the time interval and the distance from ipsilateral arms to ankles. We applied the greater value of the right and left baPWV for analysis according to the definition in other large studies (16, 17).

CIMT measurements were performed once on the far wall of the right and left common carotid arteries close to the bifurcation by using a high-resolution B-mode tomographic ultrasound system (Esaote Biomedica SpA, Italy) with a linear 7.5-MHz transducer. The distance between the leading edge of the first and the second echogenic line at the end of diastole was regarded as CIMT. We adopted the greater value of the right and left CIMT for baseline and follow-up analysis (18, 19).

Under the condition that all participants were asked to fast after dinner, blood specimens were drawn the next morning and sent for biochemical tests including fasting plasma glucose, triglycerides, apoB, LDL-C, high-density lipoprotein cholesterol (HDL-C), and total cholesterol (TC). The lipid measurements were conducted using an autoanalyzer (Modular E170; Roche, Basel, Switzerland) in the College of American Pathologists (CAP)-certified central laboratory of the study strictly following the laboratory quality control procedures. Non-HDL-C was calculated by subtracting HDL-C from TC.

### Outcome Assessment

A follow-up survey was conducted among all eligible study subjects to collect information on investigated outcomes according to the same standard protocols that were used during the baseline visit. Surrogate endpoints for cardiovascular disease, manifested by elevated baPWV, elevated PP, or elevated CIMT, were defined as the upper quartile of corresponding values at baseline or follow-up.

### Statistical Analysis

Baseline sociodemographic and biochemical characteristics were described according to different lipid statuses. Continuous data were displayed as means ± standard deviations (SD) and categorical variables expressed as numbers (percentage). Test of difference across groups was conducted by the analysis of variance (continuous variables) or chi-square test (categorical variables).

Logistic regression models were used to analyze the associations between apoB, LDL-C, and non-HDL-C and the risk of elevated baPWV, PP, or CIMT. We adjusted multivariable models with baseline age, sex, BMI, smoking status, drinking status, physical activity, glucose-lowering therapy, and lipid-lowering therapy. When apoB, LDL-C, and non-HDL-C were on a continuous scale, we chose the lowest values as a reference to visualize and detect whether there were non-linear relationships between each protein/lipid variable and outcomes by using restricted cubic splines with three knots at the 5th, 50th, and 95th percentiles.

In the discordance analysis, individuals were stratified into categories based on less than median values or greater than or equal to median values of each lipid trait. Four mutually exclusive discordance/concordance categories according to lipid variables were presented: low/low, low/high, high/low, and high/high. Discordance was defined as low LDL-C with high apoB or non-HDL-C, or vice versa. Similarly, eight categories of discordant vs. concordant values of apoB vs. non-HDL-C vs. LDL-C were obtained using the above method. The multivariable-adjusted logistic regression model was further performed to assess whether the discordant/concordant categories were in relation to the risk of elevated baPWV, PP, or CIMT.

A two-tailed *p* < 0.05 was referred to be statistically significant. We used SAS version 9.4 (SAS Institute, Cary, NC) to conduct statistical analyses and R version 4.0.5^1^ to plot restricted cubic splines and forest maps.

## Results

### Baseline Characteristics of Study Participants

Baseline characteristics of study participants are shown in Table 1. The median protein/lipid values, used as cutoffs to define discordant/concordant groups, were 95 mg/dl for apoB, 122 mg/dl for LDL-C, and 153 mg/dl for non-HDL-C in the baseline population (*n* = 5,279). There were significant differences in age, BMI, and systolic and diastolic BP across discordance/concordance groups. Among those with LDL-C below the median, 7.9 and 6.2% had a discordantly high apoB and non-HDL-C, respectively. Participants in such discordant groups more often were smokers and drinkers.

**TABLE 1:** Baseline characteristics of participants with concordant and discordant values of apoB vs. LDL-C, non-HDL-C vs. LDL-C, and apoB vs. non-HDL-C (*n* = 5,279).

															
	LDL-C < Median		LDL-C ≥ Median		*p*-value	LDL-C < Median		LDL-C ≥ Median		*p*-value	apoB < Median		apoB ≥ Median		*p*-value
	apoB < Median	apoB ≥ Median	apoB < Median	apoB ≥ Median		Non-HDL-C < Median	Non-HDL-C ≥ Median	Non-HDL-C < Median	Non-HDL-C ≥ Median		Non-HDL-C < Median	Non-HDL-C ≥ Median	Non-HDL-C < Median	Non-HDL-C ≥ Median	
															
No. of participants (n,%)	2,210 (41.9)	415 (7.9)	354 (6.7)	2,300 (43.5)		2,300 (43.6)	325 (6.2)	334 (6.3)	2,320 (43.9)		2,205 (41.8)	359 (6.8)	429 (8.1)	2,286 (43.3)	
Age (years)	56.1 ± 9.0	56.8 ± 7.8	56.8 ± 8.3	58.4 ± 7.9	<0.001	56.1 ± 9.0	56.7 ± 8.1	58.5 ± 8.4	58.1 ± 7.9	<0.001	56.1 ± 9.0	56.7 ± 8.4	58.1 ± 8.3	58.2 ± 7.9	<0.001
Male (n,%)	955 (43.2)	171 (41.2)	90 (25.4)	719 (31.3)	<0.001	994 (43.2)	132 (40.6)	96 (28.7)	713 (30.7)	<0.001	928 (42.1)	117 (32.6)	162 (37.8)	728 (31.9)	<0.001
BMI (kg/m^2^)	24.7 ± 3.2	25.6 ± 3.1	25.0 ± 3.2	25.6 ± 3.1	<0.001	24.6 ± 3.2	26.0 ± 2.9	24.6 ± 3.2	25.6 ± 3.1	<0.001	24.6 ± 3.2	25.6 ± 3.1	25.0 ± 3.3	25.7 ± 3.0	<0.001
Current smoker (n,%)	526 (25.2)	102 (25.6)	36 (10.9)	399 (18.4)	<0.001	548 (25.2)	80 (26.0)	38 (12.0)	397 (18.2)	<0.001	501 (24.1)	61 (18.3)	85 (20.7)	416 (19.3)	<0.001
Current drinker (n,%)	255 (12.2)	49 (12.6)	24 (7.1)	204 (9.3)	0.001	257 (11.9)	47 (15.2)	24 (7.6)	204 (9.3)	<0.001	239 (11.5)	40 (11.7)	42 (10.4)	211 (9.7)	0.261
Physical activity (n,%)	327 (14.8)	65 (15.7)	50 (14.1)	349 (15.2)	0.923	344 (15.0)	48 (14.8)	53 (15.9)	346 (14.9)	0.975	327 (14.9)	50 (13.9)	70 (16.3)	344 (15.1)	0.815
SBP (mmHg)	138 ± 19	142 ± 18	141 ± 19	143 ± 20	<0.001	138 ± 19	144 ± 19	141 ± 20	143 ± 20	<0.001	138 ± 19	142 ± 19	140 ± 19	143 ± 20	<0.001
DBP (mmHg)	82 ± 10	85 ± 10	84 ± 10	84 ± 10	<0.001	82 ± 10	86 ± 10	83 ± 10	84 ± 10	<0.001	82 ± 10	85 ± 10	83 ± 10	84 ± 10	<0.001
ApoB (mg/dL)	77 ± 11	101 ± 6	89 ± 5	118 ± 18	<0.001	79 ± 12	95 ± 13	96 ± 8	116 ± 19	<0.001	78 ± 11	87 ± 8	101 ± 5	118 ± 18	<0.001
LDL-C (mg/dL)	96 ± 17	112 ± 9	130 ± 8	152 ± 25	<0.001	98 ± 17	102 ± 21	127 ± 4	152 ± 25	<0.001	98 ± 17	116 ± 26	119 ± 10	151 ± 27	<0.001
Non-HDL-C (mg/dL)	127 ± 26	154 ± 22	157 ± 14	184 ± 29	<0.001	125 ± 18	174 ± 40	147 ± 5	186 ± 27	<0.001	125 ± 18	170 ± 37	144 ± 7	186 ± 28	<0.001
BaPWV (cm/s)	1529.7 ± 329.4	1599.9 ± 330.9	1550.3 ± 311.0	1630.8 ± 348.6	<0.001	1527.5 ± 328.9	1634.6 ± 327.7	1597.3 ± 361.6	1623.3 ± 342.3	<0.001	1524.1 ± 324.9	1584.3 ± 334.7	1599.5 ± 370.9	1631.1 ± 341.1	<0.001
PP (mmHg)	56.0 ± 15.0	57.7 ± 14.8	57.5 ± 15.0	59.1 ± 15.8	<0.001	56.0 ± 15.0	58.0 ± 14.5	57.8 ± 15.3	59.0 ± 15.8	<0.001	56.0 ± 14.9	57.3 ± 15.2	57.4 ± 15.5	59.1 ± 15.7	<0.001
CIMT (mm)	0.56 ± 0.10	0.58 ± 0.09	0.57 ± 0.10	0.59 ± 0.10	<0.001	0.56 ± 0.10	0.57 ± 0.10	0.58 ± 0.09	0.59 ± 0.11	<0.001	0.56 ± 0.10	0.57 ± 0.10	0.58 ± 0.09	0.59 ± 0.11	<0.001

*Data were presented as means ± standard deviations for continuous variables or numbers (percentages) for categorical variables. P-values were calculated from the analysis of variance (continuous variables) or chi-square test (categorical variables). ApoB, apolipoprotein B; BMI, body mass index; BaPWV, brachial-ankle pulse wave velocity; CIMT, carotid intima-media thickness; DBP, diastolic blood pressure; LDL-C, low-density lipoprotein cholesterol; Non-HDL-C, non-high-density lipoprotein cholesterol; PP, pulse pressure; SBP, systolic blood pressure.*

### Arterial Stiffness and Elevated Carotid Intima-Media Thickness for Lipid Traits Separately

**FIGURE 2:**
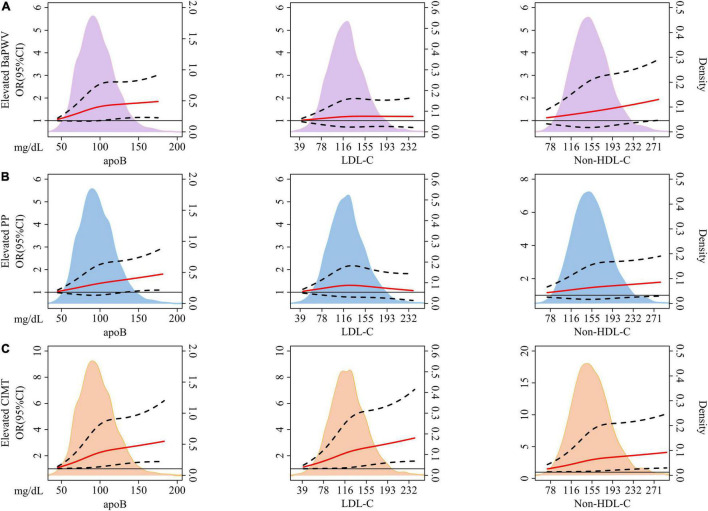
The association between baseline concentration of each lipid variable and incident elevated baPWV **(A)**, PP **(B)**, and CIMT **(C)** based on restricted cubic splines. The solid red lines represent a fitted relationship and dashed lines show 95% confidence intervals. Reference line for no association (Odds ratio: 1.0) is indicated by solid black line while areas of **purple**, **blue**, and **orange** represent the density distribution of lipid traits with incident elevated **baPWV (A)**, **PP (B)**, and **CIMT (C)** as ending points, respectively. Model was adjusted for baseline age, sex, BMI, smoking status, drinking status, physical activity, glucose-lowering therapy, and lipid-lowering therapy. ApoB, apolipoprotein B; LDL-C, low-density lipoprotein cholesterol; Non-HDL-C, non-high-density lipoprotein cholesterol; BaPWV, brachial-ankle pulse wave velocity; PP, pulse pressure; CIMT, carotid intima-media thickness; OR, odds ratio; CI, confidence interval; BMI, body mass index.

There was no non-linear relationship between each protein/lipid trait and the risk of arterial stiffness or elevated CIMT (*p* for non-linearity > 0.05). The monotonically increasing trends existed between apoB with elevated baPWV (Figure 2A), elevated PP (Figure 2B), and elevated CIMT (Figure 2C) (*p* = 0.019, 0.021, and 0.001, respectively). But LDL-C had no significant relationship with arterial stiffness (*p* = 0.555 for elevated baPWV and *p* = 0.872 for elevated PP). As for non-HDL-C, there was a significant association with elevated baPWV (*p* = 0.028), but not with elevated PP (*p* = 0.066). Both LDL-C and non-HDL-C had a strong relationship with elevated CIMT (*p* = 0.002 and 0.003, respectively).

Multivariable adjusted odds ratios (ORs) and confidence intervals (CIs) of arterial stiffness and elevated CIMT according to per SD increase in the concentration of each lipid trait were shown in Table 2. ApoB presented significant associations with all investigated outcomes. The ORs (95% CI) were 1.10 (1.02–1.20) for elevated baPWV, 1.10 (1.01–1.19) for elevated PP, and 1.19 (1.07–1.32) for elevated CIMT. Non-HDL-C was associated with elevated baPWV (OR, 1.10; 95% CI, 1.01–1.19) and CIMT (OR, 1.17; 95% CI 1.05–1.29). Notably, LDL-C showed a significant association with elevated CIMT (OR, 1.19; 95% CI, 1.07–1.32), but no relationship with elevated baPWV and PP.

**TABLE 2:** Multivariable-adjusted odds ratios of elevated **baPWV**, **PP**, and **CIMT** according to per SD increase in concentrations of apoB, LDL-C, and non-HDL-C.

	Crude OR per SD Increase	*p*-value	Adjusted OR per SD Increase	*p*-value
**Elevated BaPWV**				
ApoB	**1.19 (1.11, 1.28)**	<0.001	**1.10 (1.02, 1.20)**	0.019
LDL-C	**1.11 (1.03, 1.19)**	0.005	1.03 (0.95, 1.11)	0.555
Non-HDL-C	**1.15 (1.07, 1.23)**	<0.001	**1.10 (1.01, 1.19)**	0.028
**Elevated PP**				
ApoB	**1.22 (1.14, 1.31)**	<0.001	**1.10 (1.01, 1.19)**	0.021
LDL-C	**1.14 (1.06, 1.22)**	<0.001	1.01 (0.93, 1.09)	0.872
Non-HDL-C	**1.18 (1.10, 1.27)**	<0.001	1.08 (0.99, 1.17)	0.066
**Elevated CIMT**				
ApoB	**1.24 (1.12, 1.36)**	<0.001	**1.19 (1.07, 1.32)**	0.001
LDL-C	**1.22 (1.10, 1.34)**	<0.001	**1.19 (1.07, 1.32)**	0.002
Non-HDL-C	**1.20 (1.09, 1.31)**	<0.001	**1.17 (1.05, 1.29)**	0.003

*Crude model was an unadjusted model. Multivariable model was adjusted for baseline age, sex, BMI, smoking status, drinking status, physical activity, glucose-lowering therapy, and lipid-lowering therapy. ApoB, apolipoprotein B; BaPWV, brachial-ankle pulse wave velocity; BMI, body mass index; CIMT, carotid intima-media thickness; LDL-C, low-density lipoprotein cholesterol; Non-HDL-C, non-high-density lipoprotein cholesterol; OR, odds ratio; PP, pulse pressure; SD, standard deviations. The bold values indicated statistical significance.*

As the results presented in Figure 2 and Table 2, the superiority of apoB in predicting the risk of elevated baPWV or PP was subtle. There was necessary to further apply discordance analysis to explore the relative importance of apoB, non-HDL-C, and LDL-C, three highly correlated lipid traits, on investigated outcomes.

### Arterial Stiffness and Elevated Carotid Intima-Media Thickness for Discordant Lipid Traits

**FIGURE 3:**
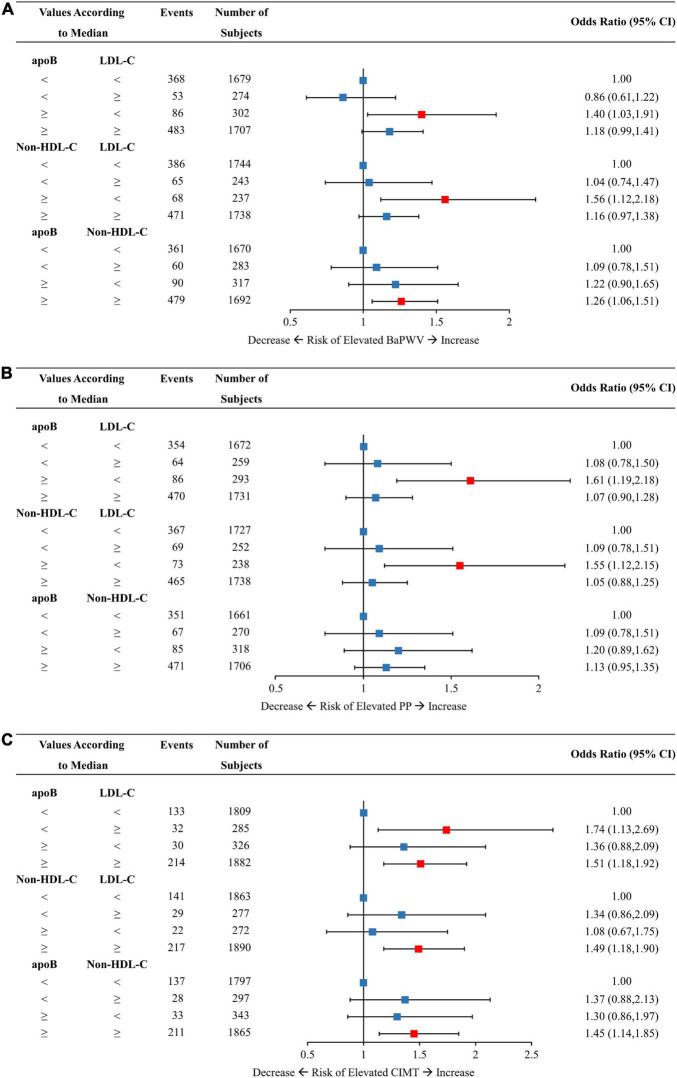
Multivariable-adjusted odds ratios of elevated baPWV **(A)**, PP **(B)**, and CIMT **(C)** by discordant vs. concordant categories of apoB, non-HDL-C, and LDL-C. The concordance/discordance categories are defined based on the medians of apoB, LDL-C, and non-HDL-C concentration levels in each analysis. We use the concordant group as reference: low apoB with low LDL-C, low non-HDL-C with low LDL-C, and low apoB with low non-HDL-C. Model was adjusted for baseline age, sex, BMI, smoking status, drinking status, physical activity, glucose-lowering therapy, and lipid-lowering therapy. ApoB, apolipoprotein B; Non-HDL-C, non-high-density lipoprotein cholesterol; LDL-C, low-density lipoprotein cholesterol; BaPWV, brachial-ankle pulse wave velocity; PP, pulse pressure; CIMT, carotid intima-media thickness; CI, confidence interval; BMI, body mass index.

**FIGURE 4:**
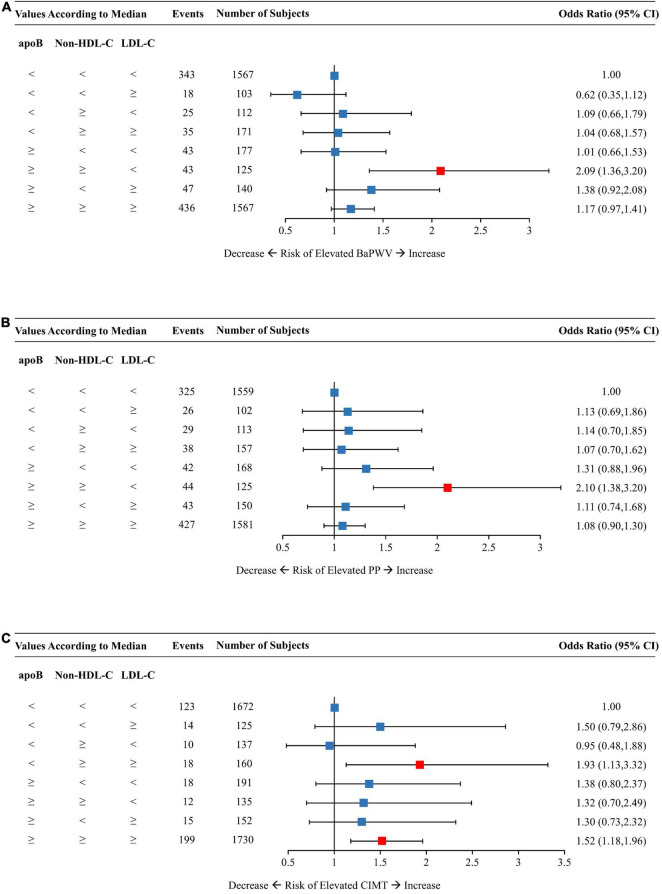
Multivariable-adjusted odds ratios of elevated baPWV **(A)**, PP **(B)**, and CIMT **(C)** by discordant vs. concordant categories of apoB, non-HDL-C, and LDL-C. The concordance/discordance categories are defined based on the medians of apoB, LDL-C, and non-HDL-C concentration levels in each analysis. The concordant group with apoB, LDL-C, and non-HDL-C all below the median is used as reference. Model was adjusted for baseline age, sex, BMI, smoking status, drinking status, physical activity, glucose-lowering therapy, and lipid-lowering therapy. ApoB, apolipoprotein B; Non-HDL-C, non-high-density lipoprotein cholesterol; LDL-C, low-density lipoprotein cholesterol; BaPWV, brachial-ankle pulse wave velocity; PP, pulse pressure; CIMT, carotid intima-media thickness; CI, confidence interval; BMI, body mass index.

Figures 3, 4 demonstrated the relationships of discordant vs. concordant categories of apoB, LDL-C, and non-HDL-C with the risk of arterial stiffness and elevated CIMT. Among those with low LDL-C, individuals with discordantly high apoB or non-HDL-C had ORs of 1.40 (95% CI, 1.03–1.91) and 1.56 (95% CI, 1.12–2.18) for elevated baPWV compared to those with concordantly low apoB or non-HDL-C, respectively (Figure 3A). For the same pattern of discordant lipid traits, the corresponding ORs (95% CI) for the risk of elevated PP was 1.61 (1.19–2.18) and 1.55 (1.12–2.15) (Figure 3B). Participants with discordantly low apoB and high LDL-C yielded a significant OR of 1.74 (95% CI, 1.13–2.69) for elevated CIMT compared to those with concordantly low apoB and low LDL-C (Figure 3C). Discordant apoB and non-HDL-C showed no association with risk of elevated baPWV, PP, or CIMT.

The concordant group with all three lipid traits below the medians constituted the reference group for analysis as shown in Figure 4. Individuals with low LDL-C but dual discordant apoB and non-HDL-C had an OR (95% CI) of 2.09 (1.36–3.20) for elevated baPWV (Figure 4A) and 2.10 (1.38–3.20) for elevated PP (Figure 4B). On the other hand, those with low apoB but dual discordant non-HDL-C and LDL-C yielded a 93% increased risk of elevated CIMT (Figure 4C). The concordant group with high levels of all three lipid traits had an OR of 1.52 (95% CI, 1.18–1.96) for elevated CIMT (Figure 4C).

## Discussion

In this prospective cohort of middle-aged and elderly Chinese adults, we observed that apoB adequately captured the risk of new-onset elevated baPWV, elevated PP, and elevated CIMT. To the best of our knowledge, the current study is the first large-scale prospective cohort among middle-aged and elderly Chinese to evaluate the associations between discordance of apoB, non-HDL-C, and LDL-C and the risk of arterial stiffness and elevated CIMT.

The prevalence of discordance of LDL-C with either apoB or non-HDL-C reached 14.6 and 12.5%, respectively. On one hand, discordant low LDL-C with high apoB or non-HDL-C presented higher risks of elevated baPWV and elevated PP compared with concordant low LDL-C and low apoB or non-HDL-C, suggesting the risk of arterial stiffness was underestimated by LDL-C alone among such discordance populations. On the other hand, elevated CIMT had a significant association with discordant high LDL-C and low apoB. Our findings suggest that while LDL-C is a good predictor for elevated CIMT, apoB, and non-HDL-C may be better indicators for arterial stiffness risk assessment.

The putative role of apoB in the course of cardiovascular events has drawn global attention. Circulating apoB is recognized as a risk predictor of CVD through the essential role in retaining atherogenic lipid particles in the arterial wall (20). In fact, several guidelines have considered apoB as the secondary target in the management of dyslipidemia and CVD risk (21, 22). Atherosclerosis and arterial stiffness, reflected by CIMT and PWV, are early stage and risk factors of CVD (23, 24). In the cardiovascular risk in Young Finns Study, apoB assessed in early life is an indicator of adulthood CIMT and PWV development (25, 26). In addition, apoB is associated with PP, a marker of arterial stiffness (27). The findings of our study support the potential value of apoB as a predictor for elevated CIMT and arterial stiffness.

To the best of our knowledge, our study is the first large-scale prospective cohort among middle-aged and elderly Chinese to explore the relationship between discordance of apoB, non-HDL-C, and LDL-C and atherosclerosis, i.e., arterial stiffness as manifested by elevated baPWV or PP, and elevated CIMT. Previous studies have shown that elevated concentrations of apoB or non-HDL-C may better reflect subclinical CVD risk than LDL-C (28, 29). The discordance analysis, affording maximal power to compare these three highly correlated lipid traits, has been widely applied in previous research. For example, in the multicenter cohort study of young adults, high apoB and low LDL-C discordance demonstrate a 55% higher risk of midlife coronary calcification than the concordance group (30). Higher levels of apoB are accompanied by higher risk irrespective of the status of LDL-C. Our findings are consistent with reports from a cross-sectional investigation composed of 402 Northern Chinese, which reveals that groups with discordance low LDL-C and high apoB or non-HDL-C are in relation to the risk of elevated baPWV (31). The risk of arterial stiffness is more strongly influenced by apoB or non-HDL-C than by LDL-C.

The explanation behind our finding that apoB or non-HDL-C may better reflect elevated baPWV or elevated PP than LDL-C is straightforward. The cholesterol contained within apoB particles is equal to non-HDL-C, comprised of the cholesterol in LDL, VLDL, IDL, and lipoprotein(a). Discordant low LDL-C with high apoB or non-HDL-C is an approximate representation of high cholesterol contained in triglyceride-rich lipoproteins (TRLs), made up of VLDL and IDL in the fasting state. Meanwhile, direct evidence exists that cholesterol within TRLs is also accumulated in the arterial wall after uptake and that populations with high TRLs are at markedly increased risk for major CVD events independent of other risk factors (32, 33). ApoB or non-HDL-C is superior to LDL-C partially owing to the extra effect of TRLs (34).

Attention is not only limited to the cholesterol content of lipid particles but also centers on the size of lipoproteins. LDL particles, which constituted about 90% of circulating apoB-containing lipoproteins, could be counted using serum apoB (35). Contextually, the size of LDL particles may present as normal in groups with concordant LDL-C and apoB. While, lipid status of low plasma LDL-C and high apoB may occur as a result of the predominance of small, dense, cholesterol-deplete LDL (30). In a cross-sectional study, very small LDL is an important contributor to elevated baPWV (36). Besides, the LDL-C (mg/dl) to ApoB (mg/dl) ratio (LDL-C/ApoB ratio), is well recognized as a representation of the size of LDL particles (37), is associated with arterial stiffness in our study (Supplementary Table 1). We divide the population into three groups according to the LDL-C/ApoB ratio and choose the highest tertile as a reference. Participants in the lowest tertile group with a smaller size of LDL particles yield significantly increased risks of elevated baPWV (OR, 1.33; 95% CI, 1.09–1.63) and elevated PP (OR, 1.54; 95% CI, 1.27–1.88). Compared to LDL, small dense LDL shows an increased atherogenic potential owing to its high affinities for arterial proteoglycans and susceptibility to oxidative modification (38, 39). Therefore, the role of small dense LDL might be another reason why apoB and non-HDL-C are better indicators for arterial stiffness risk prediction than LDL-C.

However, our study failed to demonstrate the superiority of apoB or non-HDL-C for risk assessment on elevated CIMT. The risk may be more closely related to the mass of cholesterol within LDL particles. The development of atherosclerotic plaque probably increases in a dose-dependent manner with the retention of LDL-C (40). Hence, LDL-C retains a predominant impact on elevated CIMT.

Further detection revealed that there was no difference between apoB and non-HDL-C in predicting the risk of subclinical CVD. Our findings are consistent with those inferred in several previous reports (25, 41). Koivistoinen et al. showed that the ability of apoB and non-HDL-C to detect participants with increased risk of elevated baPWV is similar (25). ApoB is the surface structural protein of each molecule of non-HDL-C. They both are markers of apoB-containing lipoproteins and are highly correlated (42). ApoB and non-HDL-C might be equivalent in assessing subclinical cardiovascular risk.

The strengths of the study include its large sample, prospective design, the accessibility of high-quality measurement of lipid parameters, and the utility of comprehensive outcomes. Nevertheless, we take cognizance of several limitations. First, the participants in this study mainly represent the middle-aged and elderly Chinese population. There is a restriction in extrapolating the results to other age groups or ethnicity. Next, the definition of discordance is arbitrary even though using the median as the cutoff point has been applied in other large studies (10, 30). Finally, elevated baPWV, PP, and CIMT, considered the observed outcomes, are surrogate indicators of cardiovascular events and measured only at baseline and follow-up. However, many studies have reported a strong relationship between the above markers and subsequent risk of CVD (11–13). And it is credible to use such markers, collected by trained study nurses according to standard protocols although measured with no repetition, as investigated outcomes in high-quality articles (14, 19).

## Conclusion

In conclusion, this prospective study showed that apoB or non-HDL-C rather than LDL-C was more strongly associated with the risk of arterial stiffness in the middle-aged and elderly population in China, but LDL-C predicted elevated CIMT well. ApoB and non-HDL-C provide utility in identifying individuals with remaining subclinical arterial stiffness burdens as LDL-C below the median. Our findings underline the importance of tackling both elevated apoB and non-HDL-C in routine clinical practice in addition to managing the LDL-C to retard or even reverse the prelude of CVD.

## Data Availability Statement

The original contributions presented in the study are included in the article/Supplementary Material, further inquiries can be directed to the corresponding author/s.

## Ethics Statement

The studies involving human participants were reviewed and approved by the Institutional Review Board of the Ruijin Hospital, Shanghai Jiao Tong University School of Medicine. The patients/participants provided their written informed consent to participate in this study.

## Author Contributions

XJ, YB, and JL contributed to the study design and concept. XJ, YQ, and RZ analyzed the data and drafted the manuscript. LL, CH, YYZ, QC, XW, HQ, RW, and YZ contributed to data interpretation and the editing of the manuscript. MX, YX, TW, ZZ, YC, ML, and WW critically revised the manuscript for important intellectual content. All authors involved in writing and revising the manuscript and had final approval of the submitted and published versions.

## Conflict of Interest

The authors declare that the research was conducted in the absence of any commercial or financial relationships that could be construed as a potential conflict of interest.

## Publisher’s Note

All claims expressed in this article are solely those of the authors and do not necessarily represent those of their affiliated organizations, or those of the publisher, the editors and the reviewers. Any product that may be evaluated in this article, or claim that may be made by its manufacturer, is not guaranteed or endorsed by the publisher.

## Footnotes

1
http://www.r-project.org
