# Supplementary material for: Discordance of Apolipoprotein B, Non-HDL-Cholesterol, and LDL-Cholesterol Predicts Risk of Increased Arterial Stiffness and Elevated Carotid Intima-Media Thickness in Middle-Aged and Elderly Chinese Adults
Source: Front Cardiovasc Med. 2022 May 18;9:906396. doi: 10.3389/fcvm.2022.906396 (PMC9157542; doi:10.3389/fcvm.2022.906396)
Supplement: Supplementary file 1 [file Table_1.DOCX]

**Supplementary Table 1.** ORs (95% CI) of elevated baPWV, elevated PP and elevated CIMT according to tertiles of the LDL-C/ApoB ratio.

|  | Tertile1  (n=1759) | Tertile2  (n=1761) | Tertile3  (n=1759) |
| --- | --- | --- | --- |
| Elevated baPWV |  |  |  |
| Cases (incidence, %) | 354 (27.57) | 332 (24.83) | 304 (22.67) |
| Model 1 | **1.30 (1.09, 1.55)** | 1.13 (0.94, 1.35) | 1(ref) |
| Model 2 | **1.37 (1.13, 1.67)** | 1.13 (0.94, 1.37) | 1(ref) |
| Model 3 | **1.33 (1.09, 1.63)** | 1.09 (0.89, 1.33) | 1(ref) |
| Elevated PP |  |  |  |
| Cases (incidence, %) | 357 (27.09) | 333 (25.15) | 284 (21.63) |
| Model 1 | **1.35 (1.13, 1.61)** | **1.22 (1.02, 1.46)** | 1(ref) |
| Model 2 | **1.52 (1.26, 1.83)** | **1.26 (1.04, 1.52)** | 1(ref) |
| Model 3 | **1.54 (1.27, 1.88)** | **1.24 (1.02, 1.51)** | 1(ref) |
| Elevated CIMT |  |  |  |
| Cases (incidence, %) | 129 (9.02) | 133 (9.46) | 147 (10.03) |
| Model 1 | 0.89 (0.69, 1.14) | 0.94 (0.73, 1.20) | 1(ref) |
| Model 2 | 0.82 (0.64, 1.06) | 0.89 (0.70, 1.15) | 1(ref) |
| Model 3 | 0.83 (0.64, 1.08) | 0.89 (0.68, 1.16) | 1(ref) |

Note: Model 1 was an unadjusted model. Model 2 was adjusted for baseline age and sex. Model 3 was adjusted for baseline age, sex, BMI, smoking status, drinking status, physical activity, glucose-lowering therapy and lipid-lowering therapy.

Abbreviations: ApoB, apolipoprotein B; BaPWV, brachial-ankle pulse wave velocity; BMI, body mass index; CI, confidence interval; CIMT, carotid intima-media thickness; LDL-C, low-density lipoprotein cholesterol; ORs, odds ratios; PP, pulse pressure.
